# Luspatercept's use in a patient with transfusion‐dependent beta‐thalassemia and intrathoracic extramedullary hematopoiesis (EMH)

**DOI:** 10.1002/ccr3.8795

**Published:** 2024-05-10

**Authors:** Mohammed Najdat Seijari, Awni Alshurafa, Mohamed A. Yassin

**Affiliations:** ^1^ Department of Internal Medicine Hamad General Hospital, Hamad Medical Corporation Doha Qatar; ^2^ Hematology department Hamad Medical Corporation Doha Qatar

**Keywords:** extramedullary hematopoiesis, luspatercept, thalassemia, transfusion‐dependent thalassemia

## Abstract

**Key Clinical Message:**

This case report and literature review examine the use of a relatively novel agent in a transfusion‐dependent beta‐thalassemia patient with extramedullary hematopoiesis (EMH). It examines the benefits and risks associated with its use and reviews the available literature while highlighting the drug's results in our patient with a higher risk profile.

**Abstract:**

Beta thalassemia can be complicated by EMH, which causes different symptoms based on location and size. Luspatercept is a new agent approved for transfusion‐dependent thalassemia and Non‐transfusion‐dependent thalassemia (NTDT). Still, its use in patients with EMH was not well studied, and literature showed an increased risk of EMH expansion or development of new masses after its use. We discuss, in this case, the results of luspatercept treatment in a patient with transfusion‐dependent thalassemia who is considered high risk for its use due to the patient's specific characteristics (history of symptomatic intrathoracic EMH, previous splenectomy, refusal to use antithrombotic medications). While also highlighting the benefits of using luspatercept regarding decreasing the iron overload and improving hemoglobin levels and examining how it was used safely to manage a transfusion‐dependent thalassemia patient with an extramedullary hematopoiesis mass with no adverse events of note.

## 1 INTRODUCTION

Extramedullary hematopoiesis (EMH) is one of the complications associated with thalassemia, with an estimated incidence of 21% in patients with Non‐transfusion‐dependent thalassemia (NTDT).^1^ Meanwhile, a recent systematic review estimated it to be evident in less than 1% of patients with transfusion‐dependent thalassemia (TDT).^2^ This dichotomy is likely related to the early aggressive management of TDT due to it being symptomatic, while there will be a delay in detecting patients with NTDT.^3^ The management of EMH was always dependent on the severity of the symptoms, the clinical condition of the patient, and the EMH location, with options including transfusion, chemotherapy with hydroxyurea (HU), surgery, and radiation therapy as the EMH is considered radiosensitive.^4^

## 2 CASE PRESENTATION

### 2.1 Case history

We present the case of a 39‐year‐old female, a known case of Thalassemia intermedia. She followed regularly with the National Center for Cancer Care and Research (NCCCR) to manage her thalassemia.

Her Surgical history is significant for a splenectomy carried out in 2004 due to hypersplenism.

Her medical history was significant for shortness of breath and persistent coughing that was diagnosed in October 2017 to be secondary to a paravertebral thoracic mass (4.5 × 3.5 × 4.3 cm) that was deemed via biopsy to be EMH (EMH). She was managed at that time with radiotherapy targeting the mass, 20GYs in 10 fractions to the T6 spine region in February 2018 in London. After this, her cough and shortness of breath symptoms improved in correlation with the mass decreasing to a size of (4 × 3 × 3 cm).

She was under recurrent supportive transfusions, a total of 11 transfusions from May 2019 to September 2021.

### 2.2 Methods‐investigations

She developed significant iron overload\severe siderosis (MRI hepatic iron overload‐ferric scan in April\2021 showed T2* time of 2 ms, corresponding to >15 mg Iron/g liver dry weight) due to the recurrent transfusions that she was receiving monthly. In addition, her antibody screening tests showed positive DAT (Poly 1+, IgG W+, C3d‐Neg) and Anti LEA positivity.

Her baseline laboratory examinations after she finished treatment for the intrathoracic EMH and before starting luspatercept (June 2021) are summarized in Table 1.

**TABLE 1:** Baseline laboratory results before starting luspatercept.

Hgb 8.6 g/dL	Crea 33 μmol\L
WBC 12.6	Urea 6.2 mmol\L
Plts 540	Na 135 mmol\L
ALT 25 μ\L	K 4.7 mmol\L
AST 24 μ\L	Ca 2.19 mmol\L
ALP 125 μ\L	Ferritin 1486 mg\L in September\2021
Bilirubin T 29.6 μmol\L	
Bilirubin D 11 μmol\L	

She was on HU 1000 mg once daily, folic acid 5 mg tablets daily, and deferasirox 1080 mg daily, in addition to regular (every 1–2 months) transfusions.

### 2.3 Treatment

Because of her transfusion‐related complication, she was started on luspatercept in September 2021 with a dose of 50 mg (1 mg\kg) subcutaneous injection every 3 weeks that she continued without modifications as she stopped needing blood transfusions (zero transfusions post‐September 2021) while also continuing her prior treatments.

**FIGURE 1:**
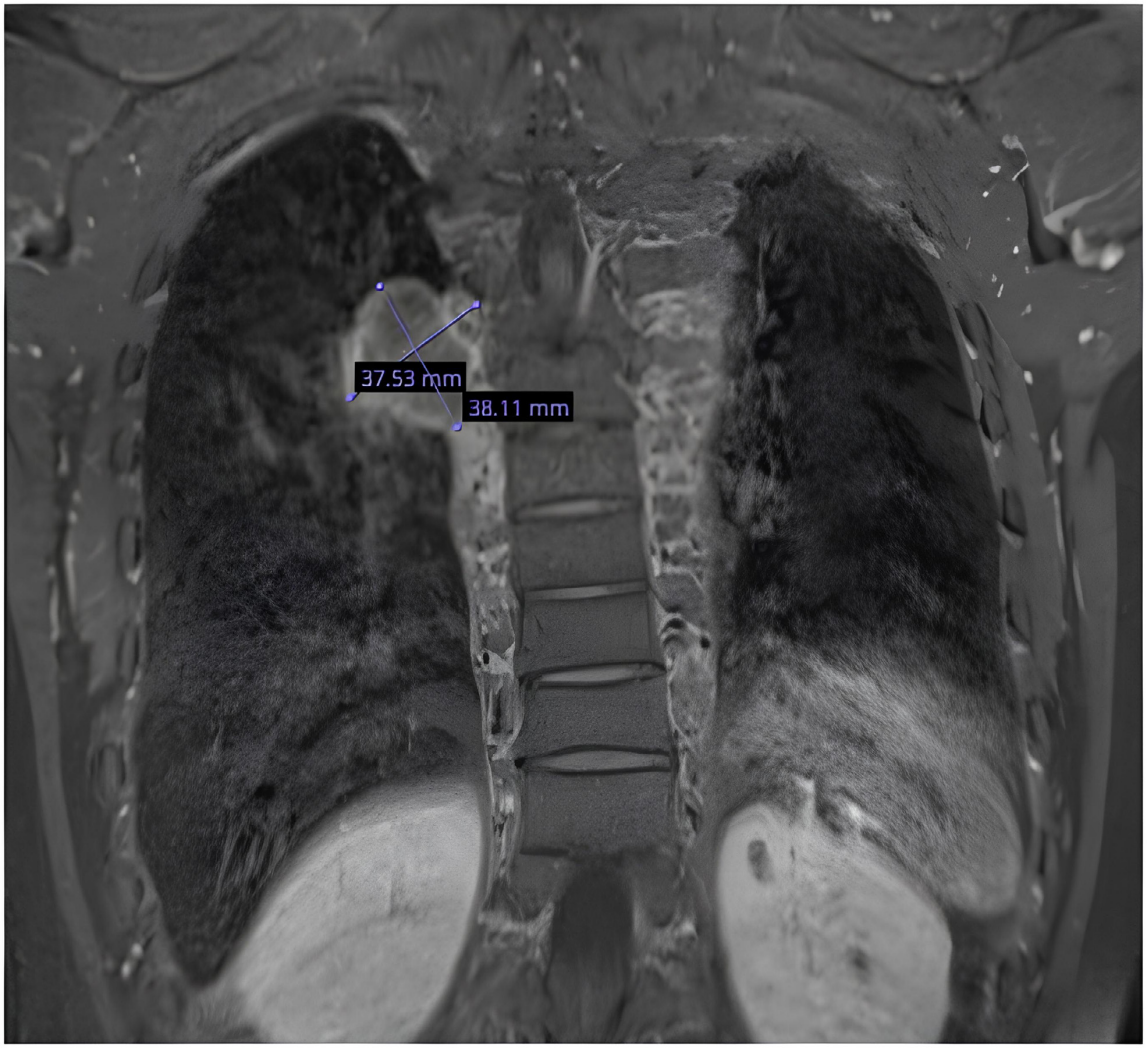
MRI thoracic spine dated April\2021.

**FIGURE 2:**
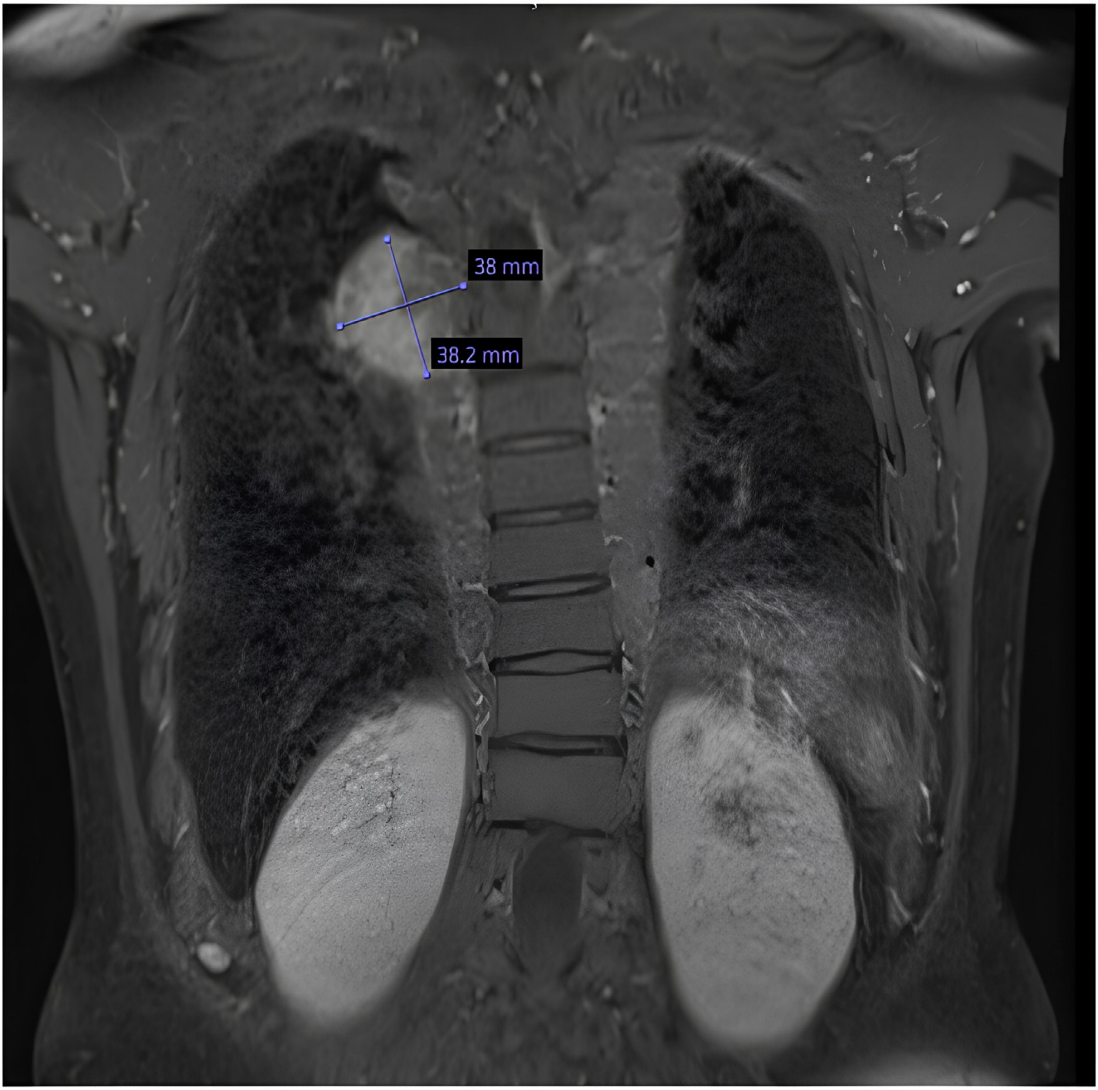
MRI thoracic spine dated October\2022.

However, due to her history of significant EMH that was symptomatic with remnant paravertebral thoracic mass, she was monitored rigorously with MRI imaging for the thoracic spine, which showed no progression or a considerable increase in the EMH mass size while she was on luspatercept treatment (Figures 1 and 2).

Although she had a splenectomy done before, she refused to start anti‐thrombosis prophylaxis after starting luspatercept due to fears of developing side effects related to aspirin.

### 2.4 Outcome and follow‐up

It was noted that she became transfusion‐independent after starting the treatment, as she didn't receive any blood transfusion for the next 18 months.

**FIGURE 3:**
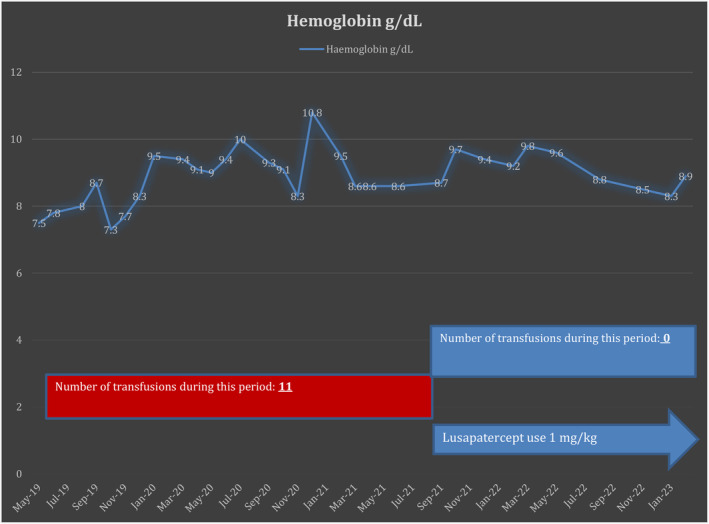
Hemoglobin level changes and number of transfusions in relation to starting the patient on lusapatercept.

Her mean hemoglobin concentration rose from 8.7 g\dL (while on regular blood transfusions) to 9.09 g\dL (off transfusions) after starting the treatment (Figure 3).

**FIGURE 4:**
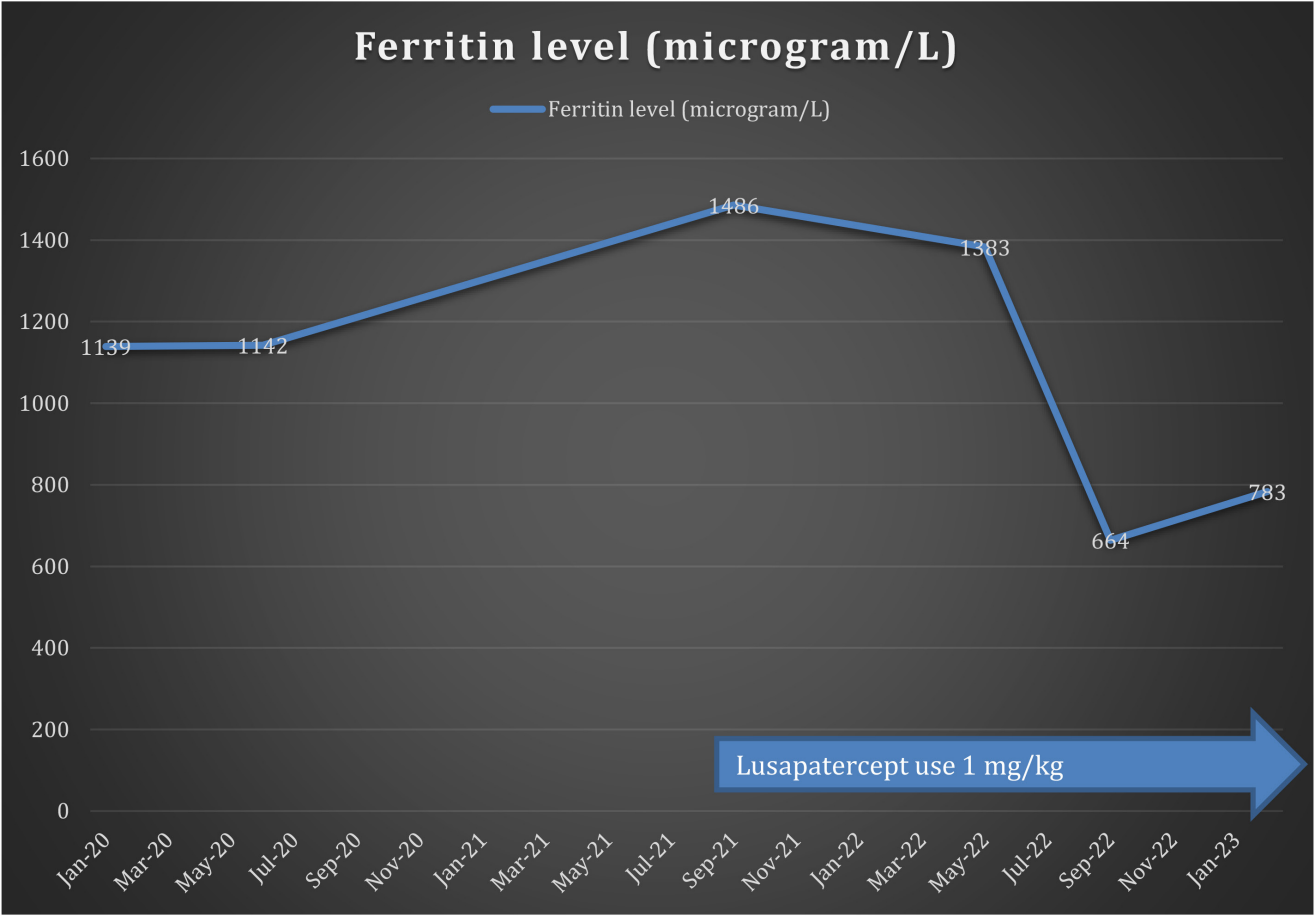
Ferritin level changes in relation to starting the patient on lusapatercept.

Her course after starting the treatment with luspatercept was uneventful from a side effects point of view and there were no documented thrombotic events. Simultaneously, her serum ferritin levels decreased from 1486 micrograms/L before treatment to an average of 950 mg/L after 16 months of therapy with Luspatercept. (Figure 4).

Our patient had a reduction in her liver iron concentration (LIC) from 24.5 mg/g (severe sideroses) as measured by ferric scan in April 2021 (before starting treatment) to 14 mg/g (moderate siderosis) based on ferric scan results in January 2023 (16 months after starting treatment) while also staying on her previous regimen of iron chelation.

She didn't show a significant increase in Heart iron concentration (HIC) on ferric scans before or after starting luspatercept.

## 3 DISCUSSION

Beta thalassemia is a well‐known inherited hemoglobinopathy that affects the production of beta hemoglobin chains, leading to alpha chain\beta chain ratio imbalance, and is classified as minor, intermedia, or major based on the severity of hemoglobin indices and analysis. It is also classified based on the need for transfusion into TDT and NTDT.^5^

Thalassemia is noted to be prevalent in special geographic distributions like the Mediterranean, throughout the Middle East, the Indian subcontinent, Southeast Asia, and the Pacific Islands, with prevalence rates of carries for beta thalassemia ranging from 1% to 20% in those areas.^6^

Long‐standing thalassemia usually gets complicated by EMH to compensate for low hemoglobin by increasing the production of erythrocytes in sites outside the bone marrow.

EMH is usually found in NTDT. However, recent reviews suggest that it is more prevalent than previously thought in TDT.^7^

EMH usually presents as soft tissue (non‐bone‐related) or para‐osseous (bone‐related) masses.^8^

Those masses can be either asymptomatic or can present with compressive symptoms related to their location, mainly in masses arising from the paravertebral region causing spinal cord or cauda equina symptoms^9^ ^,^ ^10^ or from masses in the mediastinum causing different sorts of compressive symptoms based on the location within the mediastinum (dysphagia, cough, shortness of breath, sleep apnea. etc.).^11^ ^,^ ^12^

For a long time, the cornerstone of thalassemia management was regular blood transfusion, which carried an increased risk of iron overload and alloimmunization, in addition to iron chelation therapy and HU.

The FDA approved a novel agent, luspatercept, for TDT in November 2019. It also treats anemia in patients with lower‐risk myelodysplastic syndromes with ring sideroblasts.^13^

It binds the transforming growth factor‐beta (TGF‐B) pathway ligands like Activn A, B, Growth and differentiation factor (GDF) 8 and 11, thus enhancing late‐stage erythropoiesis.^14^

The main advantage of luspatercept was decreasing the burden of transfusion dependency. It decreased the mean transfusion rate by at least 33% or 50% from baseline, increasing the interval between subsequent transfusions and relieving the patients from the cumbersome recurrent transfusions.^15^

After starting treatment, our patient didn't need any blood transfusion, while before that, she was on regular transfusion every 1–2 months.

One of the endpoints of the BELIEVE trial that examined the efficacy and safety of Luspatercept use in B‐Thalassemia patients was the aspect of iron overload due to recurrent transfusion, and it showed a reduction in iron overload as measured by serum ferritin, but with no significant change in LIC.^16^

Luspatercept is used with caution in patients with EMH, as it was noted in the BELIVE and REBLOZYL long‐term follow‐up study that in patients with TDT, 3.2% of patients developed EMH masses, and 1.9% were complicated later by the severe complication of spinal cord compression. While in patients with non‐transfusion dependent thalassemia, 6.3% of REBLOZYL‐treated patients vs. 2% of placebo‐treated patients in the double‐masked phase of the study, with spinal cord compression due to EMH masses occurring in one patient with a prior history of EMH.^17^ ^,^ ^18^

Due to the condition's rarity (EMH causing compressive symptoms) and the relatively novel nature of luspatercept, no clear guidelines are currently available to govern how to use luspatercept in those populations.

Our patient was maintained on a Luspatercept dose of 1 mg\kg for 18 months without significant change in EMH mass size on MRI spine imaging done in April 2021 (pre‐treatment) and October 2022 (posttreatment) (Figures 1 and 2). Her repeat imaging showed only a change in the intensity of the EMH mass, which could be due to iron deposition after transfusion. We believe that the stable size of EMH is likely due to the increased average hemoglobin achieved by luspatercept in addition to the maintenance treatment of HU.

Beta thalassemia major patients generally display an increased propensity to develop thromboembolic events like cerebral venous thrombosis, DVTs, and PEs. This is hypothesized to be secondary to endothelial adhesive proteins activation, monocyte and granulocyte activation, platelet activation, increased levels of coagulation factors, and decreased levels of coagulation inhibitors.^19^

It was also noted by Taher and colleagues^20^ that patients with thalassemia intermedia also show an increased risk of thrombotic/thromboembolic events, especially those who had splenectomies. Although it is advised to start thromboprophylaxis if patients with splenectomy start on luspatercept,^21^ our patient was reluctant and refused to start due to fears of aspirin side effects. Her course after starting treatment was uneventful from a thrombotic events point of view.

We believe that the prothrombotic effects of luspatercept that were previously reported in the BELIEVE trial [Thromboembolic events occurred in eight patients (3.6%) in the luspatercept arm and one patient (0.9%) in the placebo arm]^22^ and the subsequent advice provided in the prescribing information for REBLOZYL (luspatercept) should be reexamined using real‐world data now the drug has been on the market for more than 4 years, as those events could be related to the drug itself or individual factors in patients with beta‐thalassemia especially that all those events were in patients who underwent splenectomies. In addition, it should be taken into account the fact that luspatercept is not suitable in pregnancy, and some women may use oral contraceptive pills (OCPs) as a contraception method, which poses an additional thrombotic risk factor and, therefore, the actual magnitude of the thrombotic effect may be more than previously reported. We want to highlight that our patient was not using OCP while on luspatercept.

## 4 CONCLUSION

Our case showed that luspatercept could be used in a patient with beta‐thalassemia who is transfusion dependent and has EMH with caution and serial imaging to monitor for EMH expansion. Thus ensuring the patient's safety while providing the patient with the benefit of the medication by alleviating transfusion dependency and reducing the iron overload that often plagues thalassemia patients. Further large‐scale studies need to be carried out to decide on the proper frequency of monitoring for EMH and reexamine the thrombotic risk of luspatercept.

## AUTHOR CONTRIBUTIONS

**Mohammed Najdat Seijari:** Writing – original draft; writing – review and editing. **Awni Alshurafa:** Data curation; supervision; writing – original draft. **Mohamed A. Yassin:** Conceptualization; data curation; supervision.

## FUNDING INFORMATION

It will be supported by the Qatar National Library if accepted.

## CONFLICT OF INTEREST STATEMENT

On behalf of all authors, the corresponding author states that there is no financial or non‐financial conflict of interest to be declared.

## ETHICS STATEMENT

The Hamad Medical Corporation's Medical Research Centre approved the case report under the Number (MRC‐04‐23‐313).

## CONSENT

Written informed consent was obtained from the patient to publish this case report and any accompanying images.

## Data Availability

The article and supporting materials include data supporting this study. Further inquiries can be directed to the corresponding author if needed.
